# Teacher mental health literacy and its effects on helping behaviors for students with mental health problems

**DOI:** 10.1002/pcn5.70168

**Published:** 2025-08-05

**Authors:** Satoshi Yamaguchi, Jerome Clifford Foo, Tsukasa Sasaki

**Affiliations:** ^1^ Department of Physical and Health Education, Graduate School of Education The University of Tokyo Tokyo Japan; ^2^ Unit for Mental Health Promotion Research Center for Social Science & Medicine, Tokyo Metropolitan Institute of Medical Science Tokyo Japan; ^3^ Department of Psychiatry, College of Health Sciences University of Alberta Edmonton Canada; ^4^ Neuroscience and Mental Health Institute University of Alberta Edmonton Canada; ^5^ Institute for Psychopharmacology, Medical Faculty Mannheim, Central Institute of Mental Health University of Heidelberg Mannheim Germany; ^6^ Department of Genetic Epidemiology in Psychiatry, Medical Faculty Mannheim, Central Institute of Mental Health University of Heidelberg Mannheim Germany

**Keywords:** adolescent, child, helping behavior, mental health literacy, teacher

## Abstract

**Aim:**

School teachers are expected to support students with mental health problems. However, few studies have examined actual helping behaviors of teachers for the students. This study aimed to investigate the behaviors of Japanese teachers for students experiencing internalizing problems (e.g., depression/anxiety).

**Methods:**

In 2020, teachers (*n* = 465) from 48 Japanese schools (primary/junior high/senior high) answered a self‐administered questionnaire asking about: (a) the number of students in their homeroom class who seemed to have internalizing problems during the last 2–3 months, (b) whether they asked these students how they had been feeling lately, and (c) the number of students who answered “not feeling well.” Mental health literacy (MHL) in teachers was also assessed.

**Results:**

Most of the teachers (80.2%) reported that they dealt with one or more students who seemed to have internalizing problems during the last 2–3 months. Among these teachers, 94.7% had asked at least some of the students how they had been feeling, and over half of the teachers (57.8%) reported that at least one of the students answered “not feeling well.” Teachers who had confidence in helping students with depressive symptoms were more likely to recognize students who were “not feeling well” (*p* = 0.04).

**Conclusion:**

Teachers appear to be willing to help students with mental health problems when they recognize symptoms of the problems. Future studies will benefit from more closely examining whether improving confidence in teachers through MHL training increases students' willingness to disclose mental health problems to teachers, an important step in the prevention/treatment of these problems.

## INTRODUCTION

Mental health problems are highly prevalent in adolescents,^1^ but many adolescents appear reluctant to seek help for these problems when they need it.^2^ These problems can emerge as internalizing symptoms, which refer to psychological and emotional state/conditions related to withdrawal, such as depressed mood, somatic symptoms, irritability, and feeling fear about social situations.^3^, ^4^ They may also lead to other concerns in school settings such as being frequently absent from classes or school.^5^, ^6^, ^7^ Since adolescents spend much of their time in schools, school teachers are in a unique position to recognize these problems in adolescents and to appropriately support students' help‐seeking behaviors.^8^ This requires teachers to have sufficient knowledge about and positive attitudes toward mental health problems.

Knowledge about and attitudes toward mental health/illnesses were originally defined as mental health literacy (MHL).^9^ This definition has evolved to include the following MHL components, including mental health first aid skills to support others with mental health problems (ranging from basic: “talk to a person” to advanced: “listen empathically”) as well as knowledge about mental health/illnesses,^9^, ^10^ and stigma toward mental illnesses.^11^ In addition, confidence in helping students with mental illnesses has been considered as a proxy of helping behaviors for mental health problems,^12^ and has been assessed as an important outcome of MHL training programs.^12^, ^13^, ^14^

Thus far, a number of studies have examined MHL in teachers, and have observed insufficient MHL in teachers: limited knowledge about mental health/illnesses,^15^, ^16^, ^17^, ^18^, ^19^ poor recognition of specific mental illnesses,^18^, ^20^, ^21^, ^22^ and low confidence in helping students with mental health problems.^18^, ^23^ However, few of these studies have investigated teachers' actual helping behaviors for students with mental health problems.^24^, ^25^ Furthermore, no study investigating teacher MHL has examined associations between these behaviors and other components of MHL, resulting in a lack of evidence about which MHL components need to be included to develop effective MHL programs for teachers to facilitate actual helping behaviors. A few studies reported that adults (not limited to teachers) with higher knowledge, less stigma, and higher confidence had intention of and/or actually engaged in more frequent and more helpful helping behaviors for people with mental health problems.^26^, ^27^, ^28^ Exploring such associations between teacher MHL and helping behaviors for students with mental health problems may provide essential information to make MHL programs for teachers more effective.

In the current study, we aimed to investigate teachers' actual helping behaviors for students who seemed to have internalizing problems. We also aimed to examine associations between these behaviors and other components of MHL (i.e., knowledge about mental health/illnesses, stigma toward mental illnesses, and confidence in helping students with mental illnesses).

## MATERIAL AND METHODS

### Procedure and participants

Homeroom teachers from schools in Saitama Prefecture, Japan, were asked to participate in the current study. The Board of Education of Saitama prefecture (population: 7 million) asked us to make educational materials to improve MHL in teachers in the prefecture. From June to July 2020, the Board of Education informed all public schools in their jurisdiction about mental health education material that we had developed. The principals of 48 prefectural or municipal schools (20 primary schools, 18 junior high schools, and 10 senior high schools) told the Board that they wanted to use the material in their schools, and all teachers in these schools participated in the study. There were no other school selection criteria. In these schools, 465 (68.9%) homeroom teachers participated in the current study and answered the questionnaire (described below) before using the material. These homeroom teachers also participated in our previous study.^29^

### Compliance with ethical standards

The authors do not have any financial or nonfinancial conflicts of interest. The aim and contents of the study were explained to the homeroom teachers in writing, and teachers who wished to participate provided written informed consent. The study was approved by The University of Tokyo Human Research Ethics Committee (#18‐48).

### Contents of the questionnaire

Actual helping behaviors (i.e., mental health first aid skill component of MHL) of homeroom teachers for students who seemed to have internalizing problems and other components of MHL (i.e., knowledge about and attitudes toward mental health/illnesses) in teachers were assessed using a self‐administered questionnaire. The questionnaire was drafted by one of the authors (TS) and edited and refined by a team of psychiatrists, psychologists (including JCF), teachers (including SY), and school nurses. The questionnaire was written in Japanese and comprised the following three parts.

#### Part 1: Demographic variables

In the first part, the following demographic information of homeroom teachers was assessed: age, sex, school type (primary school, junior high school, or senior high school), academic degree, previous participation in mental health seminars, and experience of dealing with someone experiencing a mental illness (Table 1).

**Table 1.** Demographic data of school teachers.

Characteristics	Homeroom teachers Number (%)
Total	465
Age (years)	
20–29	163 (35.1)
30–39	169 (36.3)
40–49	63 (13.5)
50–59	51 (11.0)
60–69	19 (4.1)
No answer	0 (0.0)
Sex	
Male	249 (53.5)
Female	216 (46.5)
No answer	0 (0.0)
School type	
Primary school	162 (34.8)
Junior high school	184 (39.6)
Senior high school	119 (25.6)
No answer	0 (0.0)
Academic degree	
Associate degree^a^	36 (7.7)
Bachelor	382 (82.2)
Masters (or higher)	46 (9.9)
No answer	1 (0.2)
Previous participation in mental health seminars	
None	375 (80.6)
Once or more times	90 (19.4)
No answer	0 (0.0)
Experience of dealing with someone (e.g., students and family members) experiencing a mental illness	
No	179 (38.5)
Yes	265 (57.0)
No answer	21 (4.5)

^a^ An associate degree is an undergraduate degree in Japan awarded after a course of postsecondary study lasting 2 or 3 years.

#### Part 2: Actual behaviors of homeroom teachers for students with internalizing problems

**Figure 1.**
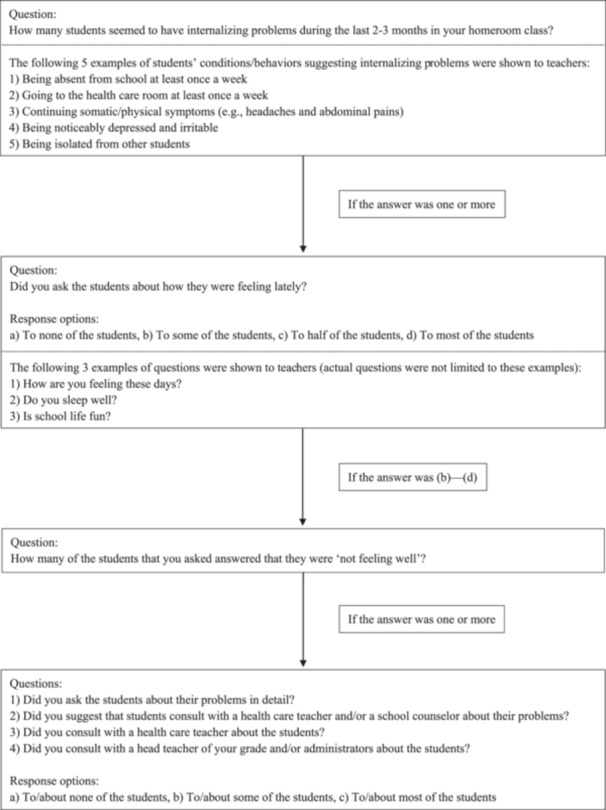
Flow chart of assessments of helping behaviors of homeroom teachers for students with internalizing problems.

In the second part, homeroom teachers' actual behaviors interacting with students having internalizing problems were assessed as shown in Figure 1. First, teachers were asked “How many students seemed to have internalizing problems during the last 2–3 months in your homeroom class?” This time frame was decided according to the timing when the current study was conducted; 2–3 months had passed after the new school year had started. Examples of students' conditions and behaviors that might suggest internalizing problems were shown to the teachers with this question (Figure 1). If the teachers answered that they had noticed such students in their classes, they were further asked, “Did you ask the students about how they have been feeling lately?” Examples of questions were shown to the teachers with this question (Figure 1). Teachers who had asked how their students were feeling were further asked “How many of these students answered ‘not feeling well’?” When teachers answered that one or more students answered “not feeling well,” they were further asked four questions about actual behaviors interacting with the students (Figure 1; Table 2).

**Table 2.** Specific helping behaviors of homeroom teachers for students who did not feel well.

Question	None of the students	Some of the students	Most of the students
Did you ask the students about their problems in detail?	3 (1.6)	32 (16.6)	156 (80.8)
Did you suggest that students consult with a health care teacher and/or a school counselor about their problems?	48 (24.9)	59 (30.6)	81 (42.0)
Did you consult with a health care teacher about the students?	37 (19.2)	40 (20.7)	113 (58.5)
Did you consult with a head teacher of your grade and/or administrators about the students?	15 (7.8)	38 (19.7)	137 (71.0)

*Note*: The sum of proportions in each row is not 100% due to missing data.

#### Part 3: Measurements of MHL

In the third part, MHL related to internalizing problems in teachers was assessed in accordance with our previous study,^18^ as below. The first section of this part comprised eight questions (Table 3) regarding knowledge about mental health/illnesses, including knowledge about the epidemiology, risk factors, and care/treatment of mental health problems/illnesses, based on previous studies^1^, ^30^, ^31^, ^32^ and the DSM‐5.^4^ The possible answers to these questions were “True,” “False,” or “I don't know.” Correct answers were scored 1 (otherwise scored 0) and the scores were added up. The score was converted from 0–8 into 0–100 (proportion of the correct answers) for ease of interpretation. In the present sample of homeroom teachers, the internal consistency (Cronbach's alpha) of the questions was 0.59.

**Table 3.** Knowledge about mental health/illnesses in homeroom teachers.

Items	Correct answer	Proportion of correct responses (%)
The incidence of most mental illnesses sharply increases in adolescence	T	55.3
About one in every 5 people will experience a mental illness in Japan	T	42.9
Students should not return to school before treatment for their mental illness has been completed	F	49.8
More than 10% of people will experience depression	T	49.2
People with mental illnesses may only have somatic symptoms, including headaches, abdominal pain, and nausea	T	71.9
The duration of treatment for depression and anxiety disorder is much more than 1 year on average	T	63.1
Due to a mental illness, people may be unable to talk to others	T	85.4
In high school students, 7 h of sleep is ideal to decrease the risk of depression	F	13.0
Average percentage of correct answers to the questions about mental health/illnesses (SD)		53.9 (23.4)

Abbreviations: F, false; SD, standard deviation; T, true.

In the second section, teachers were asked to read two case vignettes describing two teenage students with major symptoms of depression and social anxiety disorder (SAD). The vignettes were written by the authors, according to the DSM‐5 criteria of the respective disorders.^4^ The descriptions of the vignettes were as follows:

Depression: Student A goes to the health care room in the school, reporting having a headache and stomach ache, and feeling tired. Student A has trouble sleeping, does not feel like eating, does not have fun watching his/her favorite TV program, and cannot keep their mind on their studies. Student A is often late for school these days.

SAD: Student B is very shy. Student B is talkative with their family, but becomes quiet if others are there. Student B refuses to attend social gatherings with their classmates. As a result, Student B cannot make new friends, even if they want to. When giving a presentation in class, Student B is nervous, blushes, and trembles. Student B is always scared that they will do or say something embarrassing when they are around others.

Having read each vignette, teachers were asked “What is the name of the illness the student was experiencing?” for each vignette. The answer was selected from seven choices: “No illness,” “Depression,” “Schizophrenia,” “Eating disorder,” “Panic disorder,” “Social phobia,” and “I don't know.”

Four items were adapted from the “weak‐not‐sick” subscale of the Depression Stigma Scale^33^ (Table 4), which assessed beliefs that Student A in the depression vignette was weak, not ill, could control their behavior, and should be avoided. Several studies observed that the “weak‐not‐sick” stigma was associated with actual helping behaviors,^26^, ^27^ and our previous study observed that a quarter of Japanese teachers may have this stigma.^18^ One subscale item, “It is best to avoid people with a problem like Student A's so that you do not develop this problem” was modified; the item was deemed not to be appropriate for school teachers. Teachers were asked to what extent they agreed with the items. There were five answer choices (scores): “Strongly agree (1),” “Agree (2),” “Neither agree nor disagree (3),” “Disagree (4),” and “Strongly disagree (5).” The composite score was used for statistical analyses. In the present sample of homeroom teachers, the internal consistency (Cronbach's alpha) of the questions was 0.59.

**Table 4.** “Weak‐not‐sick” stigma toward depressive symptoms in the vignette among homeroom teachers.

Items	Proportions of responses (%)		
	Agree or strongly agree	Neither agree nor disagree	Disagree or strongly disagree
Student A could snap out of it if he/she wanted.	17.0	10.0	73.0
Student A's problem is a sign of personal weakness.	11.1	13.9	74.9
Student A's problem is not a real medical illness.	5.2	7.6	87.2
It is best for other students to avoid people with a problem like Student A's so that they do not develop this problem.	1.1	2.6	96.3
Proportion of teachers who agree or strongly agree with at least one of the 4 items.	26.6		

Also, teachers were asked, “How confident do you feel that you can appropriately help a student who is in a similar situation to Student A?” (student in the depression vignette). Possible answers to the question were “Fully confident,” “Confident,” “Not very confident,” and “Not confident at all.” The teacher was considered to have the confidence to help the student when their answer was “Fully confident” or “Confident.”

### Statistical analysis

Multilevel linear (for the continuous outcome) and logistic (for the dichotomous outcome) regression analyses were conducted to examine associations between actual behaviors of homeroom teachers for students who seemed to have internalizing problems (i.e., depression and anxiety) and other components of MHL related to the problems. The following components of MHL were included in the model as independent variables: knowledge about mental health/illnesses, recognition of depression and SAD, the “weak‐not‐sick” stigma toward depressive symptoms, and confidence in helping students with depressive symptoms. The effects of these variables on each of the following four outcomes were tested: (1) the number of students who teachers thought had internalizing problems (continuous outcome); (2) whether teachers asked the students who seemed to have internalizing problems about how they were feeling lately (dichotomous outcome: “to most of the students” vs. “to some, half or none of the students”); (3) the number of students who answered “not feeling well” when teachers asked about how they were feeling lately (continuous outcome); and (4) each of the four specific helping behaviors shown in Figure 1 (dichotomous outcome: “to most of the students” vs. “to some, or none of the students“). Also, all demographic variables shown in Table 1 were adjusted for, except for school type; these variables have been reported to be associated with components of MHL and/or intention of/actual behavior of helping people with mental health problems.^18^, ^24^, ^26^, ^27^, ^29^ A random effect of intercept for school was included in the analyses, since the teachers were sampled from 48 different schools. The level of significance was set at alpha = 0.05. The analyses were performed using R version 4.3.1 with lme4 and lmerTest packages.

## RESULTS

### Demographic variables

Table 1 shows the demographic data of the participants. Most of the homeroom teachers were 20–29 years or 30–39 years (71.4%) and had completed Bachelor's degrees (82.2%) as their highest education level. More than half of the teachers were male (53.5%) and previously had experiences of dealing with someone (e.g., students, family members) experiencing a mental illness (57.0%). A minority of the teachers had previously participated in a mental health seminar at least once (19.4%).

### Actual behaviors for students who seemed to have internalizing problems

**Figure 2.**
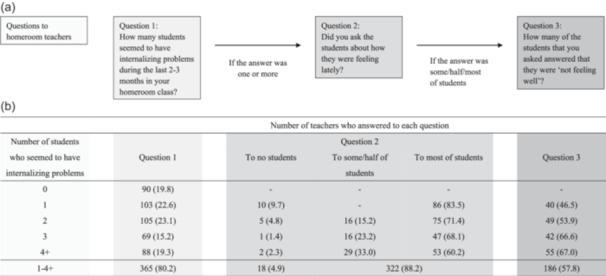
Perceived student internalizing problems. (a) Flowchart of questions and (b) corresponding responses of homeroom teachers. The sum of proportions in each row in Question 2 is not always 100% due to missing data.

Figure 2 shows the details of actual behaviors of teachers for students who seemed to have internalizing problems. In brief, most of the teachers (80.2%) answered that they had dealt with one or more students who seemed to have internalizing problems during the last 2–3 months in their homeroom class. Among these teachers, 88.2% asked at least some of the students about how they had been feeling lately, and 57.8% reported that at least one of the students answered “not feeling well.” After teachers received the answers “not feeling well“ from students, 80.8% of the teachers asked most of the students about their problems in detail, and 42.0% suggested that these students consult with a health care teacher and/or a school counselor about their problems (Table 2). Also, 58.5% of the teachers consulted with a school nurse and 71.0% consulted with a head teacher of their grade and/or administrators about most of the students (Table 2). In addition, among the teachers who thought that many (four or more) students seemed to have internalizing problems, almost all teachers (93.2%) asked at least some of the students about how they had been feeling lately. Two thirds of these teachers (67.0%) reported that at least one of the students answered “not feeling well.”

### Knowledge about mental health/illnesses, recognition of specific mental illnesses, and attitudes toward depressive symptoms

The average proportion of the correct answers to the knowledge questions about mental health/illnesses was 53.9% (standard deviation = 23.4%) (Table 3). Regarding specific questions, proportions of correct answers were low (around or below half) on five out of eight items: for example, sufficient duration of sleep lengths to decrease the risk of depression (13.0%), life‐time prevalence of mental illnesses in general (42.9%), and depression (49.2%).

Regarding the recognition of specific mental illnesses, 51.6% and 70.8% of the homeroom teachers correctly recognized depression and SAD in the respective vignettes (not shown in Tables). Less than half of the teachers (35.8%) answered that they have the confidence to help students experiencing depressive symptoms. Regarding the “weak‐not‐sick” stigma, approximately a quarter of the teachers (26.6%) agreed or strongly agreed with at least one of the four items (Table 4). Among the specific questions, the proportion of teachers who agreed or strongly agreed was the largest (17.0%) to the question “the student with depressive symptoms could snap out of it if he/she wanted.”

### Associations of homeroom teachers' actual behaviors with components of MHL and demographic variables

Table 5 shows the results of multilevel linear or logistic regression analyses. Teachers with confidence in helping students with depressive symptoms reported higher numbers of students who answered “not feeling well” when the teachers asked them about how they had been feeling lately (Unstandardized coefficient: 0.40 student, 95% confidence interval (CI): 0.02–0.77). This number did not significantly differ for other components of MHL (i.e., knowledge about mental health/illnesses, correct recognition of depression and SAD, and the ‘weak‐not‐sick’ stigma toward depressive symptoms). Teachers who correctly recognized SAD in the vignette asked about students' problems in detail (after receiving the answer “not feeling well”) significantly more frequently (Odds ratio (OR): 3.34, 95% CI: 1.20–9.30). Also, teachers with less “weak‐not‐sick” stigma toward depressive symptoms consulted significantly more frequently with a health care teacher (Odds ratio (OR): 1.18, 95% CI: 1.00–1.38) and a head teacher and/or administrators (OR: 1.21, 95% CI: 1.00–1.45) about the students. No components of MHL had significant effects on the number of students who teachers thought had internalizing problems, on whether teachers asked students who seemed to have internalizing problems about how they had been feeling lately and on whether teachers suggested that students who did not feel well should consult with a health care teacher and/or a school counselor.

**Table 5.** Effects of homeroom teacher mental health literacy on teacher and student behaviors.

Variable	Number of students who teachers thought had internalizing problems (*n* = 381)	Whether teachers asked students who seemed to have internalizing problems about how they were feeling^a^ (*n* = 289)	Number of students responding that they did not feel well when teachers asked about how they were feeling (*n* = 262)	Whether teachers asked the students about their problems in detail^b^, ^c^ (*n* = 164)	Whether teachers suggested that the students should consult with a health care teacher/school counselor^b^, ^c^ (*n* = 161)	Whether teachers consulted with a health care teacher about the students^b^, ^c^ (*n* = 163)	Whether teachers consulted with a head teacher of their grade and/or administrators about the students^b^, ^c^ (*n* = 163)
	B (95% CI)^d^	OR (95% CI)^d^	B (95% CI)^d^	OR (95% CI)^d^			
Components of MHL							
Knowledge about mental health/illnesses	0.02 (−0.09, 0.12)	0.99 (0.84, 1.17)	0.04 (−0.06, 0.13)	1.05 (0.80, 1.39)	0.96 (0.77, 1.20)	1.00 (0.81, 1.22)	0.96 (0.77, 1.21)
Recognition of depression	0.39† (−0.01, 0.78)	0.98 (0.54, 1.78)	−0.02 (−0.37, 0.34)	0.60 (0.21, 1.67)	1.20 (0.53, 2.71)	0.68 (0.31, 1.47)	1.15 (0.49, 2.68)
Recognition of SAD	−0.28 (−0.71, 0.14)	1.35 (0.71, 2.58)	−0.20 (−0.59, 0.19)	3.34* (1.20, 9.30)	1.45 (0.62, 3.41)	1.56 (0.69, 3.51)	2.11† (0.89, 5.00)
Weak‐not‐sick stigma toward DS^e^	−0.04 (−0.12, 0.05)	1.02 (0.90, 1.16)	−0.06 (−0.14, 0.02)	1.22† (0.998, 1.50)	1.11 (0.93, 1.32)	1.18* (1.00, 1.38)	1.21* (1.00, 1.45)
Confidence in helping students with DS	−0.04 (−0.45, 0.38)	0.87 (0.47, 1.63)	0.40* (0.02, 0.77)	1.11 (0.41, 2.99)	1.25 (0.57, 2.73)	1.49 (0.70, 3.17)	1.43 (0.62, 3.31)

Abbreviations: B, unstandardized coefficients; CI, confidence interval; DS, depressive symptoms; MHL, mental health literacy; OR, odds ratio; SAD, social anxiety disorder.

* *p* < 0.05.

^†^ *p *< 0.1.

^a^ “To most of the students” versus “to some, half, or none of the students.”

^b^ Teachers' specific helping behaviors for the students responding that they did not feel well when teachers asked about how they were feeling.

^c^ “To most of the students” versus “to some, or none of the students.”

^d^ Estimated values were derived from multilevel linear or logistic regression analyses with adjustment for age, sex, previous participation in mental health seminars, experience of dealing with someone experiencing a mental illness, and academic degree.

^e^ Higher score indicates lower stigma.

## DISCUSSION

The present study investigated actual behaviors of homeroom teachers from primary and junior/senior high schools for students who seemed to have internalizing problems. The teachers appeared to be willing to help their students with mental health problems (Figure 2), although their MHL needs to be improved (Tables 3 and 4). Most of the teachers asked at least some of their students about how they were feeling lately when they perceived that students seemed to be having internalizing problems, and further supported the students who were “not feeling well” (e.g., by asking the students about their problems in detail and consulting with head teachers and/or administrators [Table 2]) despite heavy workloads.^34^

Of the students who teachers were worried about and asked how they were feeling, only about half answered that they were “not feeling well” (Figure 2). The reasons for this disagreement between students and teachers may be that these students did not have actual mental health problems or that these students did not want to disclose their mental health problems to teachers. The latter is a crucial issue to deal with, considering that students can be reluctant to disclose their mental health problems,^2^ and that this reluctance could be stronger when their mental health problems become greater.^35^ Teachers may need to build trusting relationships with their students through daily communication; students may be willing to seek help from their teachers when they feel that they share a higher relationship quality (i.e., when students feel that their teachers care about them and listen to their problems).^36^ In the process of building trusting relationships, students may gradually disclose their problems when asked about recent conditions by their teachers.

Confidence in helping students with depressive symptoms was not significantly associated with whether teachers asked students who seemed to have internalizing problems about recent situations, but it was associated with the number of students who answered “not feeling well” when asked about their conditions (Table 5). These results may suggest that this confidence reflects the quality of mental health first aid skills teachers provided (e.g., not simply talking to students but also the ability to listen empathically and give support);^28^ students may be more willing to disclose concerns regarding their mental health to teachers with these skills,^36^ although this confidence was not associated with any specific helping behaviors measured in the current study for the students who were “not feeling well” (Table 5). Future studies may need to assess various aspects of teacher helping behaviors to more clearly capture the quality of first aid skills teachers provided, including assessing and assisting with any crisis, listening empathically, and giving support and information.^28^ Also, it may be possible that teachers with higher confidence tend to foster stronger relationships with students not through their first aid skills, which, in turn, may promote student disclosure. While the cross‐sectional nature of the present study limits causal interpretation, future longitudinal and/or interventional studies will benefit from examining whether improving quality of mental health first aid skills while improving confidence helping in teachers leads to willingness of students to seek help for their mental health problems from teachers.

No components of MHL had significant effects on whether teachers asked students who seemed to have internalizing problems about how they were feeling (Table 5). A reason for this may be that almost all of the teachers asked at least some of their students about how they had been feeling recently when they perceived that those students seemed to be having internalizing problems. Teachers may be willing and/or feel responsible to help students who they thought had mental health problems regardless of their MHL levels. Indeed, almost all teachers from primary and secondary schools in our previous study regarded talking to their students about mental health problems as a part of their responsibilities.^29^ While these studies were conducted in Japan, teacher attitudes may be similar across other countries; a proportion of helping behaviors for students with internalizing problems is also high in secondary school teachers in Sweden,^25^ and most teachers feel that schools should be involved in addressing mental health issues of students (in US primary and secondary schools^23^) and should play an important role in a child's mental health (in Nigerian primary schools^19^). On the other hand, we did not examine associations of MHL levels with correct recognition of internalizing problems in students, and future studies also need to investigate this association, considering that teachers may originally overlook many students with the problems; several studies reported low to moderate sensitivity of teachers' recognition of depressive (47%–75%^37^, ^38^, ^39^, ^40^, ^41^) and anxious (19%–41%^41^, ^42^) symptoms in students.

After teachers received the answer “not feeling well,” only around half of the teachers consulted with a health care teacher about most students and suggested that the students should consult with a health care teacher/school counselor (Table 2). Possible reasons for low proportions of these specific helping behaviors among teachers may be as follows. In the Japanese school health care system, mental health care is typically provided by health care teachers and school counselors/psychologists with students. However, only one health care teacher is required to be employed per school by law,^6^, ^43^ and health care teachers practice mental health care as only a minor portion of their duties, which consists mainly of regular check‐ups, providing physical/mental health first aid, and being involved in providing health education to students. Also, each school has access to a school counselor/school psychologist only for around 4 h per week on average.^6^ Accordingly, teachers may not be able to consult with these professionals when they notice that students do not feel well. This may also have led to the observed nonsignificant association between teacher MHL and frequency with which teachers encourage students who did not feel well to consult with a health care teacher/school counselors/psychologists about their problems (Table 5). In addition to improving teacher MHL, school systems for mental health care may also need to be improved (e.g., increasing numbers of and availability of health care teachers and school counselors/psychologists).

Among the components of MHL assessed, the “weak‐not‐sick” stigma toward depressive symptoms was most strongly associated with higher frequency of various specific helping behaviors that teachers engaged in (Table 5), a finding that appears to be consistent with several previous studies.^26^, ^27^ This stigma can be a target to make MHL programs for teachers more effective. Future studies need to assess whether MHL programs for teachers addressing and improving this stigma truly lead to increases in helping behaviors among teachers.

## STRENGTHS AND LIMITATIONS

A strength of the current study is that this is the first investigation of the associations between components of MHL and teacher actual helping behaviors. The results may provide useful information to improve the effectiveness of future MHL training programs for teachers to help them support students who are experiencing internalizing problems. Specifically, confidence in helping students with and the “weak‐not‐sick” stigma toward internalizing problems can be targets for making MHL programs for teachers more effective.

In contrast, the current study has several limitations. First, participants were homeroom teachers from a single prefecture in Japan. Caution may be needed to generalize the results from the current study to teachers in other prefectures and/or teachers not working as homeroom teachers, although MHL levels of the teachers in the current study were comparable to those in high school teachers in another prefecture.^18^ Second, the voluntary participation rate was not high (68.9%), although the rate was higher than that in our previous study investigating MHL in Japanese high school teachers (53.3%).^18^ Helping behaviors and MHL levels might be different between the teachers who decided to participate in the current study and those who did not. Also, the teachers were recruited through schools, where the principals decided to participate in the current study, which might also affect teacher helping behaviors and MHL levels. Third, we did not examine the actual mental health status of students; the data were based on teachers' subjective judgment and we were unable to evaluate the correct recognition of actual mental health problems in their students and its relation to MHL levels in teachers. Future studies would benefit from the inclusion of student self‐reports or, if available, clinical assessments. Fourth, due to time limitations and the request of the school board, we had to keep the assessment as short as possible. We assessed teacher helping behaviors for internalizing problems collectively, not for each illness (i.e., depression and anxiety‐related problems); we could not analyze associations between components of MHL and helping behaviors for students with potential symptoms of each disorder separately despite the possibility that helping responses differ by disorder.^26^, ^27^ While we were able to look at the “weak‐not‐sick” stigma toward and confidence in helping students with depression and their associations with teacher helping behaviors, we were not able to include these measures for SAD. Also, only four specific helping behaviors were investigated in the current study, and other potential helping behaviors were not assessed (e.g., listening empathically about student problems and giving support and information to students).^28^ In addition, specific unhelpful behaviors (e.g., listening judgmentally),^28^ which can worsen student mental health status and need to be decreased, were not assessed either. These all need to be looked at in a more comprehensive manner in future studies.

Fifth, we did not assess gender differences in teacher recognition of and helping behaviors for students who seemed to have internalizing problems. Teachers may be more likely to recognize and provide help for girls with potential internalizing problems than boys with these problems; internalizing problems are more prevalent in girls than boys, while externalizing problems, which are also common mental health problems in adolescence, including aggression, impulsivity, and conduct issues, are more prevalent in boys than girls.^44^ Future MHL programs could also address these gender differences in prevalences of internalizing and externalizing problems, which may help to reduce potential differences in teacher recognition and helping behaviors for students with different types of mental health problems. Finally, the cross‐sectional nature of the current study limits the ability to draw causal interpretations about the associations observed. Future designs may benefit from incorporating mediation analysis to examine mechanisms/temporal associations between each MHL component and teacher helping behaviors, with the hypothesis that mental health knowledge improves stigma and confidence, which then promotes helping behaviors in teachers. Additionally, later in the school year, teachers are considered to know their students better and may be able to recognize more students with potential mental health problems and to engage in more helping behaviors. Future longitudinal studies with follow‐up investigation may be able to capture these changes in teacher recognition and behaviors. These studies could also investigate whether teachers follow up with students who did not feel well.

## CONCLUSIONS

Almost all of the participating homeroom teachers asked students about recent conditions when they perceived that students seemed to be having internalizing problems, and proportions of this behavior did not significantly vary by levels of MHL in teachers, suggesting that teachers are willing to help their students regardless of their MHL levels. On the other hand, MHL in teachers was observed to be limited as in our previous study investigating MHL in Japanese high school teachers,^18^ which may lead to their difficulties in effectively supporting students with mental health problems. Developing effective MHL training programs for teachers focusing on effective first aid skills, confidence in helping students, and stigma toward mental illnesses may be needed, considering that the effectiveness of previously developed MHL programs for teachers needs to be confirmed on these outcomes.^14^

## AUTHOR CONTRIBUTIONS

Satoshi Yamaguchi and Jerome Clifford Foo drafted the manuscript, with the supervision by Tsukasa Sasaki. Satoshi Yamaguchi and Tsukasa Sasaki developed the questionnaire. Satoshi Yamaguchi analyzed the data. Tsukasa Sasaki and Satoshi Yamaguchi cooperated with the Board of Education to conduct the survey in public schools. Tsukasa Sasaki supervised the study process.

### ACKNOWLEDGMENTS

The authors are profoundly grateful to the Saitama Prefectural Board of Education for their collaboration, cooperation, and contributions to the research. This work was supported by the Japan Society for the Promotion of Science (#18H01009 and #21H00857). The sponsor had no role in the study design; the collection, analysis, or interpretation of data; the writing of the report; or the decision to submit the article for publication.

## CONFLICT OF INTEREST STATEMENT

The authors declare no conflicts of interest.

## ETHICS APPROVAL STATEMENT

The study was approved by The University of Tokyo Human Research Ethics Committee (#18‐48).

## CLINICAL TRIAL REGISTRATION

This is an observational study, and clinical trial registraion is not conducted for this study.

## PATIENT CONSENT STATEMENT

The aim and contents of the study were explained to the homeroom teachers in writing, and teachers who wished to participate provided written informed consent.

## Data Availability

The data sets generated and/or analyzed during the current study are not publicly available due to data protection and privacy regulations of the Saitama Prefectural Board of Education. They may be made available from the corresponding author on reasonable request.
